# Ameliorative Effects of Anthocyanin Metabolites on Western Diet-Induced NAFLD by Modulating Co-Occurrence Networks of Gut Microbiome

**DOI:** 10.3390/microorganisms11102408

**Published:** 2023-09-27

**Authors:** Hironobu Nakano, Kozue Sakao, Koji Wada, De-Xing Hou

**Affiliations:** 1 The United Graduate School of Agricultural Sciences, Kagoshima University, Kagoshima 890-0065, Japan; k8108910@kadai.jp (H.N.); sakaok24@agri.kagoshima-u.ac.jp (K.S.); kojiwada@agr.u-ryukyu.ac.jp (K.W.); 2 Faculty of Agriculture, Kagoshima University, Kagoshima 890-0065, Japan; 3 Department of Bioscience and Biotechnology, Faculty of Agriculture, University of the Ryukyus, Nishihara 903-0213, Japan

**Keywords:** anthocyanin metabolites, NAFLD, dyslipidemia, gut microbiome

## Abstract

Anthocyanins (Acn) have been reported to have preventive effects on Western diet (WD)-induced non-alcoholic fatty liver disease (NAFLD). However, the amount of Acn that reached the bloodstream were less than 1%, suggesting that anthocyanin metabolites (Acn-M) in the gut may contribute to their in vivo effects. This study is focused on a gut microbiota investigation to elucidate the effect of two major Acn-M, protocatechuic acid (PC) and phloroglucinol carboxaldehyde (PG), on NAFLD prevention. C57BL/6N male mice were divided into five groups and fed with a normal diet (ND), WD, WD + 0.5% PC, WD + 0.5% PG and WD + a mixture of 0.25% PC + 0.25% PG (CG) for 12 weeks. The results revealed that WD-fed mice showed a significant increase in final body weight, epididymis fat weight, liver weight and fat accumulation rate, serum total cholesterol, alanine aminotransferase, monocyte chemoattractant protein 1, and 2-thiobarbituric acid reactive substances. At the same time, these indices were significantly decreased by Acn-M in the order of PG, CG > PC. In particular, PG significantly decreased serum glucose and insulin resistance. Gut microbiome analysis revealed that PG significantly increased the relative abundance of *Parabacteroides*, *Prevotella*, *Prevotella*/*Bacteroides* ratio, and upregulated glucose degradation pathway. Interestingly, the co-occurrence networks of *Lachnospiraceae* and *Desulfovibrionaceae* in the PC and PG groups were similar to the ND group and different to WD group. These data suggest that PC and PG were able to recover the gut microbiome networks and functions from dysbiosis caused by WD. Therefore, PG might act as a master metabolite for anthocyanins and prevent WD-induced NAFLD and gut dysbiosis.

## 1. Introduction

Nonalcoholic fatty liver disease (NAFLD) is hepatic steatosis due to excessive accumulation of free fatty acids (FFA) secreted by lipolysis from adipose tissue [1]. Abnormal FFA secretion leads to lipotoxicity and ultimately to cytotoxicity and even cell death [2]. The prevalence of NAFLD is accelerating worldwide, with an estimated 32% of the world’s population affected [3]. The gut microbiome consists of more than several thousand species and provides a variety of benefits disadvantages to the host’s health, such as the maintenance of membrane barrier integrity, bile acid metabolism, nutrient acquisition, and prevention of pathogen invasion [4]. The relationship between the gut microbiome and the development of NAFLD was suggested by the finding that fecal microbiota transplantation caused NAFLD in germ free mice [5]. In addition, gut dysbiosis due to Western diet is thought to promote liver damage through the production of toxic metabolites (ethanol, saturated fatty acids, polyamines, hydrogen sulfide, etc.) in the gut [4].

While a lot of research on gut microbiome has focused on the association between disease and the composition of specific microorganisms, a recent co-occurrence network in gut microbiome analysis studies showed an essential role of microbial interactions in disease progression [6]. Co-occurrence networks in the gut microbiome are complex interactions among bacteria in which species cooperate and compete for nutrients and metabolites [7]. The keystone species in co-occurrence network are also excellent target candidates for gut-based interventions because they are defined as species necessary for ecosystem integrity and stability [8]. Gut microbiome network analysis in NAFLD patients showed key species such as *Porphyromonas loveana*, *Alistipes indistinctus*, and *Dialister pneumosintes*, as potential targets for appropriate intervention strategies for NAFLD treatment. Gut microbiome network analysis in NAFLD patients showed key species such as *Porphyromonas levii*, *Alistipes indistinctus*, and *Dialister pneumosintes* [9]. Moreover, *Porphyromonas levii*, *Alistipes indistinctus*, and *Dialister pneumosintes* were potential targets for appropriate intervention strategies for NAFLD treatment [9].

A number of studies have reported that bioactive compounds, especially anthocyanins (Acn), one of the flavonoids, may have great potential for the prevention and treatment of NAFLD [1]. Our previous study revealed that administration with bilberry Acn powder (BA) containing 36% improved Western diet (WD)-induced NAFLD symptoms in mice [10]. In this case, BA lowered the levels of serum total cholesterol (T-Cho), low-density lipoprotein cholesterol (LDL-c), liver fat content, 2-thiobarbituric acid-reactive substances (TBARS), and increased the relative abundance of gut *Akkermansia muciniphila* and *Parabacteroides* [10].

On the other hand, the blood reach of Acn has been reported to be less than 1%, suggesting that anthocyanins metabolites (Acn-M) may contribute to their in vivo effects [11]. Acn is stable in the stomach (pH = 1.3 ± 0.2) as a glycoside and is detected in the gastric epithelium, then absorbed by sodium-dependent glucose cotransporters (SGLT1), glucose transporters, and monocarboxyl transporters [11]. In the small intestine (pH = 8.2 ± 0.2), Acn is degraded to anthocyanidins by lactase fluoridine hydrolase and β-glucosidase derived from epithelial cells and intestinal bacteria [11,12]. Cyanidin as a representative anthocyanidin was degraded to equal amount of protocatechuic acid (PC) and phloroglucinol carboxaldehyde (PG) under neutral pH without microbiome [13]. On the other hand, in the presence of the microbiome, anthocyanidins were degraded to PC, gallic acid (GA), syringic acid (SA), vanillic acid (VA), ferulic acid (FA), 4-hydroxybenzoic acid (HBA), hippuric acid (HA), PG, coumaric acid (COA), and 2-hydroxy-4methoxybenzoic acid (HMBA) [14,15]. Acn are absorbed via SGLT1 while PC and PG, which are more hydrophobic than Acn, and are absorbed passively through the biological membrane [11].

The preventive effect of some Acn-M on NAFLD has been investigated. Oral administration of PC (10 mg·kg^−1^·day^−1^) attenuated the serum levels of alanine aminotransferase (ALT), T-Cho, LDL-c, triglycerides (TG), glucose, and liver fat accumulation in high fat and high cholesterol diet-induced NAFLD in male C57/BL6 mice [16]. Oral administration of PG (5 mg·kg^−1^·day^−1^) reduced the glucose levels in serum, TG, T-Cho, and hepatic steatosis in male C57/BL6 mice fed with a high fat diet (HFD) [17]. Oral administration of 0.2% GA ameliorated impaired lipid homeostasis in a mouse NAFLD model induced by HFD-streptozotocin (STZ) [18]. Oral administration of 0.05% SA reduced body weight, visceral fat mass, serum levels of leptin, tumor necrosis factor-α, interferon-γ, interleukin-6, monocyte chemoattractant protein 1 (MCP-1), insulin resistance (IR), hepatic lipid content, droplets, and early fibrosis [19]. Oral administration of VA (10 mg·kg^−1^·day^−1^) decreased serum levels of ALT and LDL-c in HFD-induced obese mice [20]. Oral administration of HMBA (0.2 mg·kg^−1^·day^−1^) inhibited hyperglycemia in streptozotocin-induced diabetic rats [21], and also suppressed serum T-Cho, TG, LDL-c, and liver fat accumulation in chronic ethanol treatment rats [21]. However, oral administration of FA (20 mg·kg^−1^·day^−1^) in a HFD mouse model showed no preventive effect on hyperlipidemia [22]. 

These reports suggest that Acn-M may have preventive effects of NAFLD. However, the relative effects of Acn-M on the gut microbiome and NAFLD remains unclear. To fully understand the mechanisms of Acn-M on the prevention of NAFLD, two major Acn-M, PC and PG, as well as their mixture, were orally administrated in WD-induced NAFLD mice. Then the metabolic markers and gut microbiome, and especially the co-occurrence network in the gut microbiome, were investigated in this study.

## 2. Materials and Methods

### 2.1. Chemicals and Reagents

Lard and cellulose were purchased from Sigma-Aldrich Co., LLC. (Tokyo, Japan). Soybean oil, cholesterol, choline bitartrate, methionine, fructose, 1,1,3,3-tetraethoxypropane, and 2-thiobarbituric acid were purchased from Nacalai Tesque, Inc. (Kyoto, Japan). AIN-93G mineral mix and AIN-93G vitamin mix were purchased from Oriental Yeast Co., Ltd. (Tokyo, Japan). PC and PG were purchased from Tokyo Chemical Industry Co., Ltd. (Tokyo, Japan). Corn starch was purchased from Sanwa Starch Co., Ltd. (Nara, Japan). Edible Acid Casein 30–60 Mesh was purchased from (Meggle, Wasserburg am Inn., Germany). Sucrose was purchased from Introduction of Hayashi Pure Chemical Ind., Ltd. (Osaka, Japan). Hexane was purchased from FUJIFILM Wako Pure Chemical Co. (Osaka, Japan).

### 2.2. Animal Experiment Design

**Figure 1:**
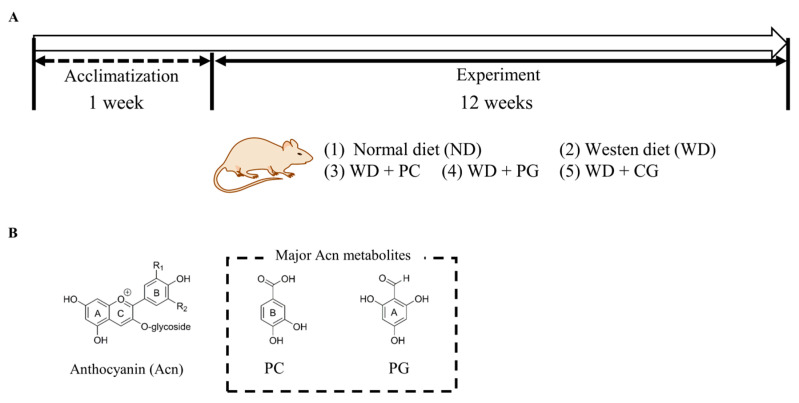
Schematic diagram of animal experiment. (**A**) Duration and grouping of mice in animal experiments. We designed 0.5% PC and 0.5% PG group as well as CG 0.25% PC + 0.25% PG (CG) group in mice diet. (**B**) A structure of Acn, PC, and PG. PC: protocatechuic acid, PG: phloroglucinol carboxaldehyde.

The animal experiment protocol was drafted according to the guidelines of the Animal Care and Use Committee of Kagoshima University (Permission NO. A12005). Male C57BL/6N mice (5 weeks old) from Japan SLC Inc. (Shizuoka, Japan) were housed separately in cages with wood shaving bedding, under controlled light (12 h light/day) and temperature (23.5 °C), and free access to water and feed. After acclimatizating for one week, the mice were randomly divided into the following five groups (n = 5): normal diet (ND) group, Western diet (WD) group, WD + 0.5% PC group, WD + 0.5% PG group, and WD + 0.25% PC + 0.25% PG (CG) group (Figure 1 and Table A1). ND contained 3% lard and 3% soybean oil; WD contained 30% lard, 3% soybean oil, and 1.5% cholesterol. Normal water was provided to ND group, and 4% fructose water was provided to WD groups. Mice were sacrificed at 18 weeks of age after overnight fasting.

### 2.3. Measurement of Serum Biochemical Indexes

Blood was obtained from the mice’s orbital veins and collected into a tube with coagulant (separable microtubes, FUCHIGAMI, Kyoto, Japan) for 30 min at room temperature. The sera were acquired with centrifugation at 4000 rpm for 5 min and stored at −80 °C until use. The serum levels of ALT, T-Cho, and glucose were measured with an automated analyzer for clinical chemistry (SPOTCHEM EZ, Arkray, Kyoto, Japan). The level of LDL-c was calculated using the Friedewald equation (LDL-c = T-Cho − HDL-c − TG/5) [23]. The insulin and MCP-1 serum concentrations were measured with an ELISA kit (Invitrogen, San Diego, CA, USA) according to the manufacturer’s instructions. The homeostatic model assessment index for IR was calculated with the function of fasting glucose × fasting insulin/405 [24].

### 2.4. Measurement of 2-Thiobarbituric Acid Reactive Substances (TBARS)

The liver TBARS concentration was measured according to the previous study [10,25]. The concentration of the TBARS was expressed in nmol/mg liver proteins. The range of 10–100 μM of 1,1,3,3-tetraethoxypropane was used as standard.

### 2.5. Measurement of Hepatic Lipid

The hepatic lipid was measured according to the previous study [10]. Briefly, 100 mg of liver was homogenized in 500 μL of hexane, supernatant evaporated, and the residue was weighed.

### 2.6. Gut Microbiome Analysis by 16S rRNA Gene Sequencing

The feces DNA was extracted by FastDNA SPIN kit for Feces (MP Bio Japan K. K., Tokyo, Japan). The composition of gut bacterial communities was analyzed by sequencing 16S rRNA genes as described in our previous paper [10,26]. The sequences were grouped in amplicon sequence variants (ASVs) with 97% similarity by QIIME 2.0.

### 2.7. Prediction of Functional Abundances Based on 16S rRNA Gene Sequences by PICRUSt2

PICRUSt2 (Phylogenetic Investigation of Communities by Reconstruction of Unobserved States, version 2.5.2) is a software for predicting functional abundances based only on marker gene sequences [27]. In brief, ASVs were normalized for total 16S rRNA gene copy number followed by metagenome predictions (PICRUSt2) against MetaCyc (Metabolic pathways from all domains of life) database (https://metacyc.org/ (accessed on 1 May 2023)).

### 2.8. Co-Occurrence Network Analysis in Gut Microbiome

Correlation network analysis in the gut was performed using a correlation method of pairwise Sparse Correlations for Compositional data (SparCC) [28] between taxonomic features. The community in co-occurrence networks were analyzed by the cluster_louvain method [29], using igraph package (version 1.2.11) in R (version 4.1.3). The node betweenness centrality [30] normalized to z-value was calculated by igraph package in R. A co-occurrence network was drawn by Cytoscape (version 3.9.1). In the correlation network, nodes represent taxa of the gut microbiome, and edges represent correlations greater than the correlation threshold (i.e., 0.3) between pairs of taxa.

### 2.9. Statistical Analysis

Results are expressed as means ± standard error of the mean. Significant differences between the groups were determined using one-way ANOVA followed by Tukey’s test (IBM SPSS Statistics 27, IBM Japan, Ltd., Tokyo, Japan). A probability of *p* < 0.05 was considered significant.

## 3. Results

### 3.1. Organ Weight and Indexes of Lipid and Glucose

**Figure 2:**
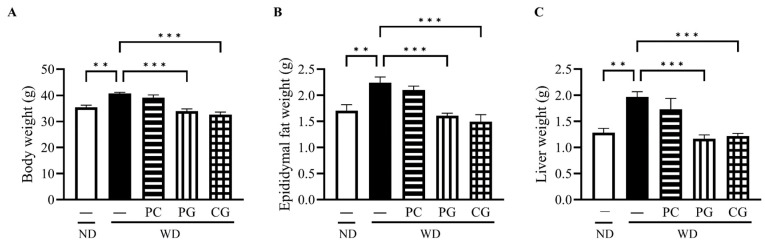
Effects of Acn-M on organ weight. (**A**) body weight, (**B**) epididymis fat weight, and (**C**) liver weight at 18 weeks. These data represent the mean ± SE of five mice for each group. Columns with asterisk differ significantly (** *p* < 0.01, *** *p* < 0.001 in Tukey test). ND: normal diet, WD: Western diet, PC: 0.5% PC, PG: 0.5% PG, CG: 0.25% PC + 0.25% PG.

**Figure 3:**
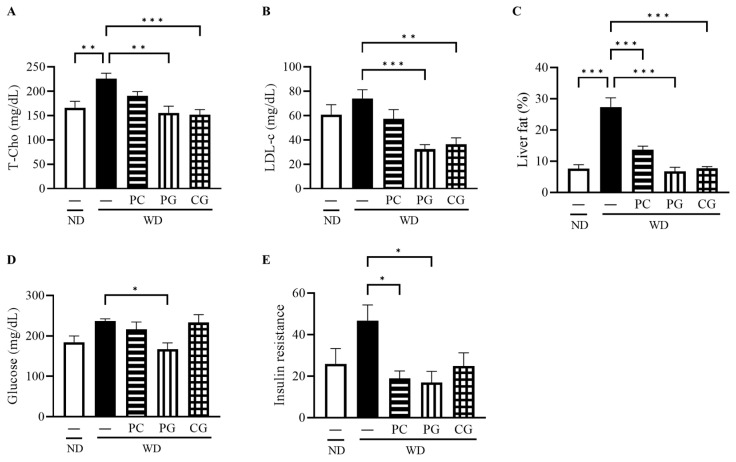
Effects of Acn-M on glucose/fat metabolism. (**A**) serum T-Cho, (**B**) serum LDL-c, (**C**) liver fat, and (**D**) serum glucose, and (**E**) insulin resistance at 18 weeks. These data represent the mean ± SE of five mice for each group. Columns with an asterisk differ significantly (* *p* < 0.05, ** *p* < 0.01, *** *p* < 0.001 in Tukey test). ND: normal diet, WD: Western diet, PC: 0.5% PC, PG: 0.5% PG, CG: 0.25% PC + 0.25% PG. T-Cho: total cholesterol, LDL-c: low density lipoprotein cholesterol.

The final body weight (BW, Figure 2A), epididymis fat weight (EFW, Figure 2B), and liver weight (LW, Figure 2C) in the WD group at 18 weeks were all significantly higher than the ND group. Supplementation with PG and CG significantly decreased WD-increased BW, EFW and LW (Figure 2A–C). In lipid metabolism-related markers, serum T-Cho, serum LDL-c, and liver fat were significantly increased in WD group compared to the ND group (Figure 3A–C). T-Cho and LDL-c were significantly decreased in the PG and CG groups (Figure 3A,B). Liver fat was significantly reduced in all supplementation groups compared to the WD group (Figure 3C). In glucose metabolism-related markers, serum glucose was significantly decreased in the PG group, and insulin resistance was significantly decreased in the PC and PG group compared to the WD group (Figure 3D,E).

### 3.2. Liver Damage, Inflammation, and Oxidative Stress

**Figure 4:**
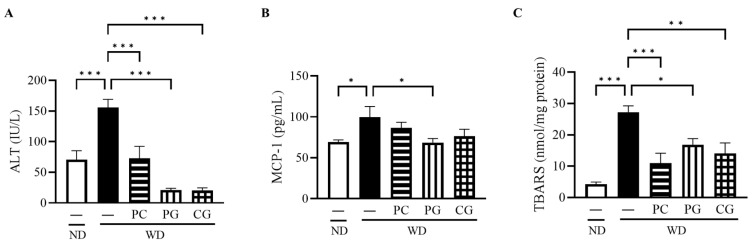
Effects of Acn-M on liver damages, inflammation, and oxidative stress indicators. (**A**) serum ALT, (**B**) serum MCP-1, and (**C**) liver TBARS at 18 weeks. These data represent the mean ± SE of five mice for each group. Columns with asterisk differ significantly (* *p* < 0.05, ** *p* < 0.01, *** *p* < 0.001 in Tukey test). ND: normal diet, WD: Western diet, PC: 0.5% PC, PG: 0.5% PG, CG: 0.25% PC + 0.25% PG. ALT: alanine aminotransferase, MCP-1: monocyte chemotactic protein-1, and TBARS: 2-thiobarbituric acid reactive substances.

The serum ALT, a liver deviation enzyme, was significantly decreased in all treatment groups compared to the WD group (Figure 4A). The serum level of MCP-1, an inflammatory cytokine, was significantly reduced in the PG group compared to the WD group (Figure 4B). To evaluate the oxidative status in the liver, we measured TBARS, a secondary product of lipid peroxidation that has been widely adopted as a sensitive assay method for lipid peroxidation. Liver TBARS was significantly increased in the WD group compared to the ND group and significantly decreased in the PC, PG, and CG groups (Figure 4C).

### 3.3. Gut Microbiome Structure Analysis by 16S rRNA Gene Sequencing and MetaCyc Pathway Analysis by Picrust2

**Figure 5:**
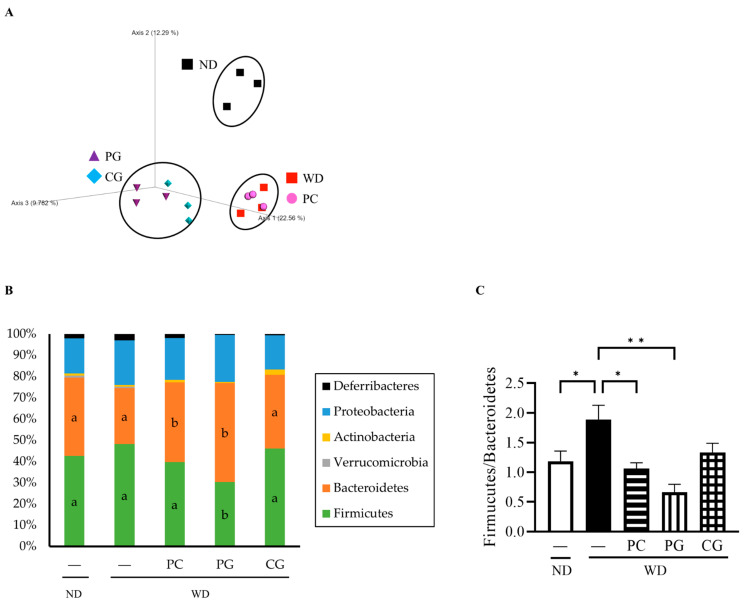
Effects of Acn-M on the gut microbiome. (**A**) The species compositions of gut microbiomes were assessed by β-diversity analyses using the principal coordinate analysis (PCoA) of Jaccard. Each dot represents the experiment’s ending point (18 weeks) from each group. Modulation of the gut microbiome at the phylum level (**B**) and Firmicutes/Bacteroidetes ratio (**C**). The column represents the mean ± SE from each group. Columns with different letters (*p* < 0.05 in Tukey test) and asterisk (* *p* < 0.05 and ** *p* < 0.01 in Tukey test) changed significantly. ND: normal diet, WD: Western diet, PC: 0.5% PC, PG: 0.5% PG, CG: 0.25% PC + 0.25% PG.

The relative abundance of gut bacteria in all groups was analyzed by 16S rRNA gene sequencing. The similarities in gut microbiome structure among the groups were estimated by principal coordinate analysis (PCoA) plots based on Jaccard. As shown in Figure 5A, the structure of the gut microbiome was divided into the following three groups: (1) ND, (2) WD and PC, and (3) PG and CG.

**Figure 6:**
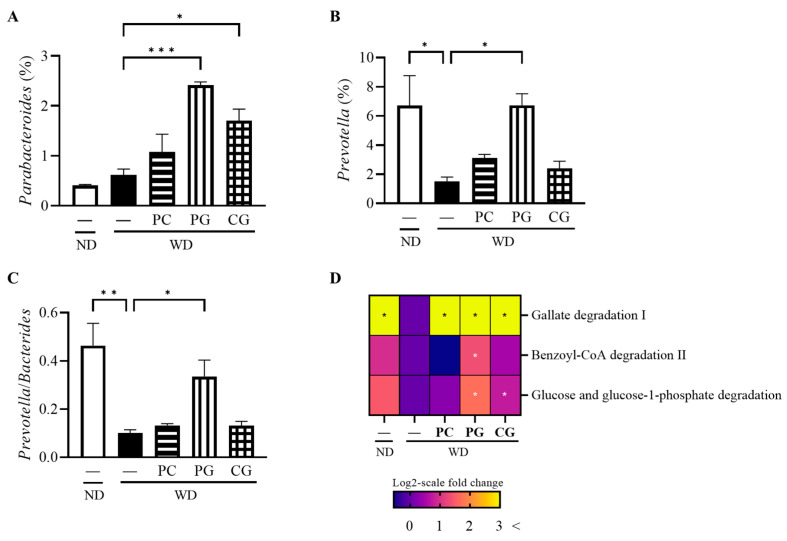
Modulation of gut bacteria at the genus level and MetaCyc pathway and gene by Acn-M in the gut microbiome. (**A**) *Parabacteroides*, (**B**) *Prevotella*, and (**C**) *Prevotella*/*Bacteroides*, and (**D**) heatmap of MetaCyc pathway and gene expression levels. In the heat map, the values were calculated as log2-scale fold change compared to the WD group. As a result, the values in the WD group were all zero, while positive values indicated an increase, and negative values indicated a decrease compared to the WD group. Columns and heatmap with asterisk differ significantly (* *p* < 0.05, ** *p* < 0.01, and *** *p* < 0.001 in Tukey test). ND: normal diet, WD: Western diet, PC: 0.5% PC, PG: 0.5% PG, CG: 0.25% PC + 0.25% PG.

Consequently, the changes in individual microbial species were investigated at the phylum level (Figure 5B). In comparison to the WD group, the relative abundance of *Firmicutes* was significantly decreased while the relative abundance of *Bacteroidetes* was significantly increased in the PC and PG groups. Thus, the *Firmicutes/Bacteroidetes* ratio (F/B) was significantly decreased in the PC and PG groups compared with the WD group (Figure 5C). Moreover, PG and CG significantly increased the relative abundance of *Parabacteroides* (Figure 6A). PG significantly increased the relative abundance of *Prevotella* (Figure 6B) and *Prevotella*/*Bacteroides* ratio (Figure 6C).

Finally, based on 16S rRNA gene sequencing data, we predicted metagenomic pathways against the gene families present in MetaCyc that are precalculated in PICRUSt2 (Figure 6D). Gallate degradation I pathway was increased in all Acn-M groups and Benzoyl-CoA degradation II pathway was increased further in the PG group compared to the WD group, which were belong to phenols ring-cleavage dioxygenase. Glucose and glucose-1 phosphate degradation pathway was increased in the PG group.

### 3.4. A Co-Occurrence Network Analysis in the Gut Microbiome

**Figure 7:**
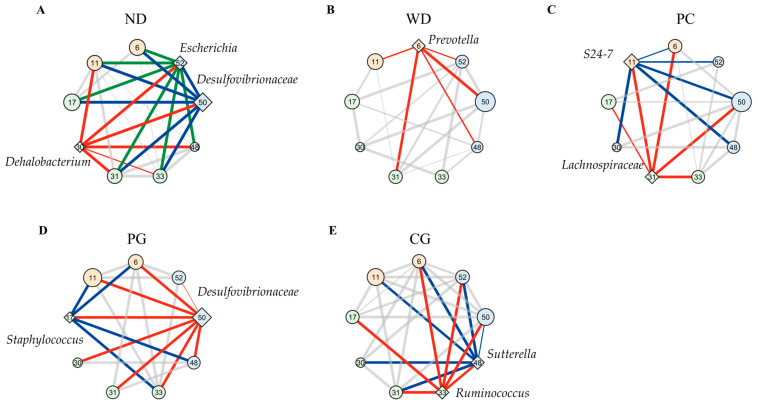
Modulation of co-occurrence network analysis in the gut microbiome. Co-occurrence gut microbiome networks in (**A**) ND, (**B**) WD, (**C**) PC, (**D**) PG, and (**E**) CG group were performed using SparCC correlations between taxonomic features. These nodes show each bacterium, which belongs to the phyla of Firmicutes (green), Bacteroidetes (orange), and Proteobacteria (blue). The diamond shape of nodes means keystone bacteria (z > 2) and circle shape (z < 2) means other bacteria according to z value of betweenness centrality normalized to z-value. The larger the size of the nodes, the greater the abundance presented. These edges show over 0.3 correlations, and the thicker edge, the higher correlation. Red, blue, and green edges lead from high centrality of taxa (diamond nodes) to other nodes. These nodes were displayed keystone bacteria and bacteria whose abundance were more than fourth rank. The numbers in node mean 6: *Prevotella*, 11: *S24-7*, 17: *Staphylococcus*, 30: *Dehalobacterium*, 31: f_*Lachnospiraceae*; _, 33: f_*Lachnospiraceae*; g_[*Ruminococcus*], 46: *Allobaculum*, 48: *Sutterella*, 50: f_*Desulfovibrionaceae*; g_, and 52: *Escherichia*. ND: normal diet, WD: Western diet, PC: 0.5% PC, PG: 0.5% PG, CG: 0.25% PC + 0.25% PG.

A co-occurrence network analysis in the gut microbiome was inspired by the report of altered interaction between intestinal bacteria and the progression of liver cirrhosis [31]. Figure 7 showed co-occurrence network in the gut microbiome performed by SparCC between taxonomic features. Figure 7 displayed keystone bacteria and bacteria whose abundance bacteria were more than a fourth. The community of frequently coexisting taxon (nodes in the same background color belong to the same community) consisted of the 3, 4, 2, 3, and 2 communities in the ND, WD, PC, PG, and CG networks, respectively. To evaluate the topological properties of each taxon within the network, we calculated the betweenness centrality (Table 1). A taxonomy with high betweenness centrality is expected to play important topological roles in interconnecting pairs of other taxa in co-occurrence network [32]. We set taxonomy with a betweenness z-value greater than two as the keystone bacteria in the network as previous study [33].

The results suggested that *Dehalobacterium*, f_*Desulfovibrionaceae*; g_, and *Escherichia* were keystone bacteria in the ND group (Figure 7A and Table 1). f_*Desulfovibrionaceae*; g_ and *Escherichia* belonged to same community and phylum of Proteobacteria. These two keystone bacteria co-occurred bacteria species (bind to green and blue edges) were matched by 89% in the ND group. *Prevotella* was the only keystone bacterium in the WD group (Figure 7B), and the fourth largest betweenness (z-value = 1.69) was in the ND group (Table 1). The keystone bacteria were *S24-7* and f_*Lachnospiraceae*; _ in the PC group, *Staphylococcus* and f_*Desulfovibrionaceae*; g_ in the PG group, and [*Ruminococcus*] and *Sutterella* in the CG group (Figure 7C–E and Table 1). Two keystone bacteria co-occurred bacteria species (bind to red and blue edges) were matched 50, 61, and 67% in the PC, PG, and CG groups, respectively. 

**Figure 8:**
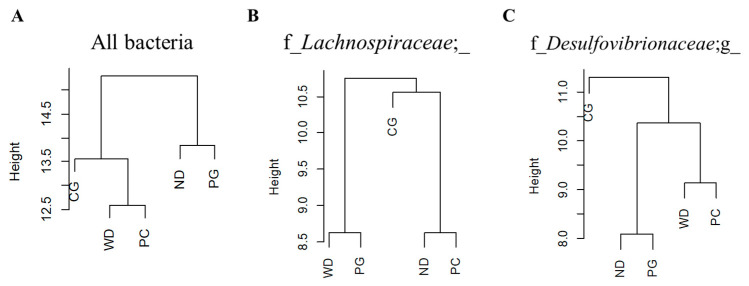
Dendrogram of using betweenness centrality and SparCC correlations. The dendrogram of (**A**) all bacteria, (**B**) f_*Lachnospiraceae*; _, and (**C**) f_*Desulfovibrionaceae*; g_ with edges in the gut microbiome network. Cluster analysis of the bacterial species that were co-occurring with each keystone bacteria in the gut microbiome network showed that the groups formed different clusters. ND: normal diet, WD: Western diet, PC: 0.5% PC, PG: 0.5% PG, CG: 0.25% PC + 0.25% PG.

Next, we analyzed the edges possessed by specific bacteria to estimate the role of each bacterium in the gut microbiome network. The dendrogram was drawn by cluster analysis based on the betweenness centrality (node) and SparCC correlations (edge thickness) for all bacteria in co-occurrence network, the ND and PG groups belonged to the same cluster (Figure 8A). The edges with f_*Lachnospiraceae*, which was one of the keystone bacteria in the PC group, were classified in the same group as ND and PC through cluster analysis (Figure 8B). Similarly, the edges with f_*Desulfovibrionaceae*; g_, which was one of the keystone bacteria in the PG group, was classified into the same group in the ND and PG groups (Figure 8C). In addition, the edges with *Ruminococcus* and *Sutterella*, which were one of the keystone bacteria in the CG group, were not similar to the ND, WD, PC, and CG groups.

## 4. Discussion

### 4.1. The Preventive Effect of Acn-M on Mice NAFLD Symptom

Acn is degraded to anthocyanidins in the gut [11,12], then anthocyanidins are further degraded to PC, GA, SA, VA, FA, HBA, HA, PG, COA, and HMBA [14,15]. In this study, the major Acn-M, PC and PG, revealed preventive effects on WD-induced NAFLD by modulating serum biochemical markers, liver oxidative stress markers, and the gut microbiome structure and network. In another study similar to our study concept comparing Acn-M and PC, there was a weaker anti-hyperlipidemia and stronger anti-inflammatory effect than GA and COA [34]. However, there were no studies comparing the effects of PC and PG on metabolic syndrome as far as we know. 

In this study, serum levels of ALT and LDL-c, important serum markers of NAFLD, were attenuated by all Acn-M groups, but the preventive effect of PC on NAFLD was weaker than PG. This is possibly due to the differences in the metabolic processes of PC and PG. It has been reported that PC is more easily metabolized than PG, and the amount of PG in blood at 48 h was approximately twice that of PC [14]. Thus, the weaker effect of PC on NAFLD prevention in this study may be related to the differences in the metabolic properties of PC and PG.

Our data revealed that PG, but not PC, significantly decreased serum glucose. A previous study has observed a significant decrease in glucose with 0.1% PC [34], although there was no significant difference in α-glucosidase inhibitory activity between cyanidin, PC, and PG [35]. On the other hand, plasma glucose level was increased in the NAFLD model with HFHC, but not changed in only HFD model [36]. These data suggested that HFHC model, which is similar to our study, induced stronger hyperglycemia than the HFD model alone. It is possible that the lack of glucose-lowering effect of 0.5% PC in this study may be attributed to the stronger hyperglycemia induced by HFHC. PG (20 mg/kg body weight) and PG metabolite, HMBA (0.2 mg·kg^−1^·day^−1^) were also reported to have anti-hyperglycemic effect in the STZ-induced diabetes SD rats [21,37], which explains the lowered effect of PG in this study.

Oxidative stress is considered to be one cause of NAFLD [38], and the antioxidant activity of Acn-M might contribute to the preventive effects of NAFLD. The 50% inhibitory concentration in the 1,1-diphenyl-2-picrylhydrazyl (DPPH) radical scavenging assay was 66.9 μM and 75.9 μM in PC and PG, respectively [35]. Moreover, PC metabolite’s antioxidant capacity in vitro are weaker than PC’s. The order of the DPPH radical scavenging capacity was PC > FA > VA, that of superoxide radical scavenging capacity was PC > FA > VA, and that of reducing power was PC > FA > VA [39]. In our study, liver TBARS as oxidative stress in vivo was attenuated by PC, PG and CG. However, the effect of PG was weaker than that of PC and CG, which was the same trend as DPPH radical scavenging assay.

### 4.2. The Effect of Acn-M on Some Specific Gut Microbiome and Metagenomic Pathways

The changes in the gut microbiome were reported in diet consisting of PC, but there is no unified view. One study reported to increase *S24-7* and decrease *Lactobacillaceae* and F/B ratio [40], and another study reported to increase *Desulfovibrio* and decrease *Prevotella* [41]. In this study, F/B ratio, which is increasing in obese people [42], was reduced by PC as previous study [40], but other bacteria were not significantly altered.

There are no studies on the relationship between PG and the gut microbiome. Our data first revealed that PG could increase several relative abundances of interesting bacteria such as *Parabacteroides* and *Prevotella*. *Parabacteroides distasonis* gavage alleviated obesity and metabolic dysfunctions via production of succinate and secondary bile acids [43], which support the higher preventive effect of PG on NAFLD than PC in this study. In addition, *Prevotella copri* gavage exhibited anti-glycemic effect [44] and *Prevotella* was decreased in the NAFLD patients group than the healthy group [45]. These data may support why PG decreased serum glucose in this study.

It is noticed that *A. muciniphila* disappeared in all Acn-M groups. In contrast, Acn significantly increased relative abundance of *A. muciniphila* [10,46]. Additionally, *Prevotella* was increased in the PG group but disappeared by Acn [10]. *A. muciniphila* and *Prevotella* commonly had anti-glycemic effect [44,47] and mucin degradation enzymes [48,49]. Therefore, both Acn and PG can prevent NAFLD due to the proliferation of *A. muciniphila* and *Prevotella*, respectively, and both bacteria have similar roles.

Different results have shown the effect of Acn and Acn-M on the growth of *Akkemansia*. Cyanidin-3-O-glucoside (7.2 mg/kg BW/day) as Acn and PC (2.3 mg/kg BW/day) as Acn-M did not have a proliferative effect on *Akkermansia* abundance in the HFHS mouse model [50]. The mixtures (40 mg/kg BW) of delphinidin-3-O-glucoside, cyanidin-3-O-glucoside, and peonidin-3-O-glucoside as Acn increased abundance of *Akkermansia* in caecum contents of HFD mice [51]. Ten-day intake of proanthocyanidin (360 mg/kg BW/day), a polymer of anthocyanidin, increased the growth of *Akkermansia* in the gut microbiome of mice [52]. Thus, the proliferative effect of *Akkermansia* in the gut may depend on the amount of Acn in feed, and not on PC and PG as Acn-M.

The metagenomic pathways of the gut microbiome predicted by PICRUSt2 showed glucose metabolism were specifically changed in the Acn-M group. Under anaerobic conditions, the gut microbiome degrades most compounds with benzene rings via the intermediate benzoyl-CoA, leading to the formation of acetyl-CoA [53]. PC and PG are compounds with a phenolic group bound to the benzene ring and are metabolized by the gut microbiome. In this study, the gallate degradation I pathway was increased in all Acn-M groups, while the benzoyl-CoA degradation II pathway was increased only in the PG and CG groups, suggesting that the gut microbiome was able to metabolize Acn-M.

Focusing on glucose metabolism in metagenomic pathways of the gut microbiome, glucose and glucose-1 phosphate degradation pathway, which converts glucose to gluconate, increased only in the PG group. Gluconate is finally metabolized to pyruvate and glyceraldehyde 3-phosphate in the Entner–Doudoroff pathway of the gut microbiome [54]. Therefore, it is possible that the increase in the glucose and glucose-1 phosphate degradation pathway upregulated glucose consumption, resulting in decreased serum glucose in the PG group of mice.

### 4.3. The Effect of Acn-M on Co-Occurrence Network of the Gut Microbiome

The mechanisms of NAFLD and non-alcoholic steatohepatitis (NASH) pathogenesis are not fully understood. Research on network analysis of the entire gut microbiota, not just the increase or decrease in specific bacterium, has been recently conducted for NASH pathogenesis [55]. In this study, network analysis of gut microbiome revealed that a characteristic network and the keystone bacteria differed in each group. *Prevotella* was the keystone bacterium in gut microbiome of the WD group. Similar to our study, *Prevotella buccalis* was the keystone bacteria in obesity and NASH in humans [9]. In addition, abundance of *Prevotella* was decreased in progression of NASH in human [9] and NASH in mice induced by STZ-HFD [56]. In contrast, the gut microbiome network had another keystone bacterium different from *Prevotella* in ND and Acn-M groups. Thus, it is possible that Acn-M reduced the effect of *Prevotella* on the gut microbiome network to prevent NAFLD progress.

Although the gut microbiomes in the PC, PG, and CG groups were not significantly different from the WD group, some gut microbiome networks with keystone bacteria (e.g., f_*Lachnospiraceae*; _ and f_*Desulfovibrionaceae*; g_ were similar to the ND group (different to the WD group) in the PC and PG groups. *Lachnospiraceae* can utilize lactate and acetate to produce butyrate [57], which ameliorates hepatic steatosis and inflammatory mediators such as MCP-1 [58]. *Desulfovibrionaceae* was reported to produce hydrogen sulfide and cause inflammation [59]. The metabolic functions possessed by keystone bacteria in the PC and PG groups may have similar effects on symbiotic bacteria as the ND group, therefore, PC and PG are able to recover gut microbiome networks and functions from dysbiosis caused by WD. On the other hand, the edges of gut microbiome networks with *Ruminococcus*, produced alcohol and caused hepatic inflammation [60], was not similar to ND, WD, PC, and CG groups. In other words, PC and PG ameliorated abnormal network changes rather than having a strong effect on specific bacteria.

## 5. Conclusions

Acn-M, in the order of PG, CG > PC, prevented an increase in hepatic fat accumulation rate, serum LDL-c, ALT, MCP-1, and TBARS of lipid peroxides caused by WD. In particular, PG significantly increased the relative abundance of *Parabacteroides*, *Prevotella*, *Prevotella*/*Bacteroides* ratio, glucose degradation pathway in gut microbiome, resulting in a decrease in serum glucose. Moreover, co-occurrence networks of *Lachnospiraceae* and *Desulfovibrionaceae* in the PC and PG groups were similar to the ND group and different to the WD group, respectively. These data suggest that PC and PG were able to recover the gut microbiome networks and functions from WD-induced dysbiosis. Therefore, PG might act as a master metabolite of anthocyanins to prevent WD-induced NAFLD and gut dysbiosis.

## Figures and Tables

**Table 1:** List of betweenness centrality (z-value > 2) in gut microbiome network.

z-Value	ND	WD			
	−	−	PC	PG	CG
f_[*Paraprevotellaceae*]; g_[*Prevotella*]	1.69	**3.45**	−0.55	0.07	0.13
f_*S24-7*; g_	0.78	−0.07	**2.24**	0.14	1.65
f_*Staphylococcaceae*; g_*Staphylococcus*	−0.78	−0.32	0.12	**2.03**	−0.35
f_*Dehalobacteriaceae*; g_*Dehalobacterium*	**3.84**	−0.65	−1.47	−0.41	−0.22
f_*Lachnospiraceae*;_	0.24	0.69	**3.05**	0.43	1.12
f_*Lachnospiraceae*; g_[*Ruminococcus*]	0.41	−0.46	1.22	0.14	**2.97**
f_*Alcaligenaceae*; g_*Sutterella*	−0.75	−0.62	−0.89	−0.08	**2.70**
f_*Desulfovibrionaceae*;g_	**2.51**	−0.26	0.82	**4.40**	0.48
f_*Enterobacteriaceae*; g_*Escherichia*	**2.38**	0.23	0.37	−0.41	0.09

ND: normal diet, WD: Western diet, PC: 0.5% PC, PG: 0.5% PG, CG: 0.25% PC + 0.25% PG. Bold numerical values are larger than two.

## Data Availability

The data presented in this study are available in the article.

## Abbreviations

Acn	anthocyanins
Acn-M	anthocyanins metabolites
ALT	alanine aminotransferase
BA	bilberry Acn powder
BW	body weight
CG	0.25% PC + 0.25% PG
DPPH	1,1-diphenyl-2-picrylhydrazyl
EFW	epididymis fat weight
FA	ferulic acid
FFA	free fatty acids
GA	gallic acid
HA	hippuric acid
HBA	4-hydroxybenzoic acid
HFD	high fat diet
HMBA	2-hydroxy-4methoxybenzoic acid
IR	insulin resistance
LDL-c	low-density lipoprotein cholesterol
LW	liver weight
MCP-1	monocyte chemoattractant protein 1
MetaCyc	metabolic pathways from all domains of life
NAFLD	nonalcoholic fatty liver disease
NASH	non-alcoholic steatohepatitis
ND	normal diet
PC	protocatechuic acid
PCoA	principal coordinate analysis
PG	phloroglucinol carboxaldehyde
PICRUSt2	Phylogenetic Investigation of Communities by Reconstruction of Unobserved States
SA	syringic acid
SGLT1	sodium-dependent glucose cotransporters
SparCC	Sparse Correlations for Compositional data
STZ	streptozotocin
TBARS	2-thiobarbituric acid-reactive substances
T-Cho	total cholesterol
TG	triglycerides
VA	vanillic acid
WD	Western diet

## Appendix A

**Table A1:** Dietary compositions of each group.

Components (%)	ND	WD			
	−	−	PC	PG	CG
Lard	3	30	30	30	30
Soybean oil	3	3	3	3	3
Corn Starch	45	16.5	16	16	16
Casein	20	20	20	20	20
Sucrose	20	20	20	20	20
Cellulose	4	4	4	4	4
Mineral Mix	3.5	3.5	3.5	3.5	3.5
Vitamin Mix	1	1	1	1	1
Cholesterol	0	1.5	1.5	1.5	1.5
Choline bitartrate	0.2	0.2	0.2	0.2	0.2
Methionine	0.3	0.3	0.3	0.3	0.3
PCA (PC)			0.5		0.25
PGA (PG)				0.5	0.25
Total calories (kcal/100 g)	374	517	515	515	515

ND: normal diet, WD: Western diet, PC: 0.5% PC, PG: 0.5% PG, CG: 0.25% PC + 0.25% PG.
